# Effects of Caste on the Expression of Genes Associated with Septic Injury and Xenobiotic Exposure in the Formosan Subterranean Termite

**DOI:** 10.1371/journal.pone.0105582

**Published:** 2014-08-20

**Authors:** Claudia Husseneder, Dawn M. Simms

**Affiliations:** Department of Entomology, Louisiana State University Agricultural Center, Baton Rouge, Louisiana, United States of America; The Ohio State University/OARDC, United States of America

## Abstract

As social insects, termites live in densely populated colonies with specialized castes under conditions conducive to microbial growth and transmission. Furthermore, termites are exposed to xenobiotics in soil and their lignocellulose diet. Therefore, termites are valuable models for studying gene expression involved in response to septic injury, immunity and detoxification in relation to caste membership. In this study, workers and soldiers of the Formosan subterranean termite, *Coptotermes formosanus,* were challenged by bacterial injection or by no-choice feeding with a sublethal concentration (0.5%) of phenobarbital. Constitutive and induced expression of six putative immune response genes (two encoding for lectin-like proteins, one for a ficolin-precursor, one for the Down syndrome cell adhesion molecule, one for a chitin binding protein, and one for the gram-negative binding protein 2) and four putative detoxification genes (two encoding for cytochrome P450s, one for glutathione S-transferase, and one for the multi antimicrobial extrusion protein), were measured via quantitative real time polymerase chain reaction and compared within and among 1) colonies, 2) treatment types and 3) castes via ANOVA. Eight genes were inducible by septic injury, feeding with phenobarbital or both. Colony origin had no effect on inducibility or differential gene expression. However, treatment type showed significant effects on the expression of the eight inducible genes. Caste effects on expression levels were significant in five of the eight inducible genes with constitutive and induced expression of most target genes being higher in workers than in soldiers.

## Introduction

As social insects, termites (order Blattaria, formerly Isoptera [1]) live in densely populated colonies with a specialized caste system characterized by morphological differentiation and division of labor between worker, soldier and reproductive castes. Relatedness within termite colonies is usually high since members of all castes are the offspring of a limited number of reproductives (kings and queens [2]). Morphological and behavioral differentiations among castes are therefore based on gene expression rather than genotype [3].

Termites must have evolved strong and versatile defense mechanisms against toxic xenobiotics and pathogens in adaptation to living in densely populated colonies of closely related individuals, with intense social interactions in nest conditions conducive to microbial growth and transmission as well as being exposed to contaminants in the soil, insecticides and a lignocellulosic diet containing secondary metabolites produced by plants. Detoxification enzymes to metabolize xenobiotics are therefore essential. Previous studies using purification and biochemical characterization showed that termites possess multiple cytochrome P450s [4], glutathione S-transferases (GSTs [4]) and microsomal esterases [5] and show different levels of susceptibility to termiticides [6], [7], [8]. However, the relationship between constitutive or inducible gene expression and enzyme activity/insecticide susceptibility has not yet been established.

While detoxification enzymes protect insects from toxic effects of xenobiotics, the innate immune system is the first line of defense against microbial infections. The innate immune system is primarily non-specific and serves to recognize invading pathogens, activate humoral and cellular immune responses leading to inactivation and clearance of pathogens.

The insect innate immune system recognizes invading pathogens through an array of pattern recognition receptors (PRR) that bind to conserved microbial cell membrane or wall epitopes serving as immune elicitors, such as lipopolysaccharides (Gram-negative bacteria), peptidoglycans (Gram-positive bacteria) and β-1,3-glucan (fungi). Insect PRRs include C-type lectins, gram-negative bacteria binding proteins, peptidoglycan-, lipopolyssaccharide-and β-1,3-glucan binding protein, and β-1,3-glucan recognition protein [9], [10], [11]. Binding of microbial immune elicitors by PRRs triggers cellular and humoral immune responses. Cellular defense consists of a variety of hemocytes responsible for sealing wounds via aggregation [12], phagocytosis, encapsulation of invading microbes and nodulation, which leads to melanization by the prophenol oxidase (PPO) pathway and sequestration [13], [14]. Humoral response includes the release of antimicrobial peptides (AMPs) into the hemolymph mainly from the fat body and the activation of proteases, which trigger melanization via the PPO pathway ([15], and references therein). The PPO pathway also produces intermediates that are directly toxic to microorganisms (e.g., quinones).

While the PPO pathway initiates melanization and produces antimicrobial compounds within minutes, recognition of foreign cell wall components also triggers intracellular signaling pathways that work within a couple of hours to days. The Toll pathway responds mainly to Gram-positive bacteria and fungi, while the immune deficiency (IMD) pathway reacts predominantly upon infection with Gram-negative bacteria. Both pathways result in expression of PRRs and antifungal peptides and/or AMPs. The Toll pathway also triggers activation of serine proteases involved in melanization. Other signaling pathways (JNK- and JAK/STAT) are triggered by exposure to lipopolysaccharides of Gram-negative bacteria, but their contribution to insect immunity is less well researched [16].

Termites possess both humoral and cellular immunity (reviewed in [17]). Pioneering work by, e.g., [18], [19] showed that immunization via sub-lethal dosages of pathogens induces the production of AMPs in the termite hemolymph. A number of genes and peptides related to this kind of humoral immunity have been described in termites. Among them are a transferrin gene which was up-regulated following exposure to an entomopathogenic fungus [20], termicin, spinigerin [18] and gram-negative binding proteins (GNBPs) which can act as both PRRs and AMPs in several termite species [21]. Production of AMPs appears to differ not only between species [22], but also between termites belonging to different colonies or castes [17], [19], [23]. The components of cellular immunity in termites are less well studied although melanization and encapsulation of foreign bodies have been frequently used as bioassay for immune competence (e.g., [24]).

While both, detoxification enzymes as well as immune proteins have been detected in termites, full genome screening and gene expression studies are still in their infancy where termites are concerned. We chose the Formosan subterranean termite (FST), *Coptotermes formosanus* Shiraki (Blattodea: Rhinotermitidae), as model to investigate constitutive and induced gene expression related to xenobiotic metabolism and immunity in termites. This termite represents one of the most successful and economically important invasive pest species worldwide [25], [26]. As a subterranean termite, *C. formosanus* forages and nests mainly in the soil and is associated with urban environments and as such exposed to xenobiotics not only through their natural diet (wood) but also through soil contaminants and insecticides. Colonies are densely populated and can contain over a million individuals [27]. Such a life style should be conducive for growth and spread of pathogens, however, 50 years of failed biocontrol efforts [28] suggests the existence of strong synergistic immune and detoxification defense mechanisms of subterranean termite colonies.

Annotation of our previously constructed expressed sequence tag library of the FST revealed the presence of putative detoxification and immune-related genes [29]. Out of those, we selected the 10 genes with the most similarity to known detoxification genes (e.g., those encoding cytochrome P450s, glutathione S-transferase) and immune genes (e.g., those encoding for gram-negative binding proteins, lectin- and fibrinogen-like peptides as well as the Down syndrome cell adhesion molecule [29]). A recent study aiming at a more complete description of the immune related transcriptome in *C. formosanus* [30] confirmed the presence of those genes among hundreds of putatively immune-related genes encoding for PRRs, signal modulators, transductors and effector molecules (such as AMPs) whose expression was upregulated after fungal and/or bacterial challenge. However, colony or caste specific gene expression, inducible detoxification or possible crosslinks between genes involved in immunity and xenobiotic metabolism were not considered. In fact, very few studies, have considered differential gene expression of immunity and detoxification genes among castes of termites [31], [32]. Therefore, the principal goals of our study were to identify genes associated with immunity and/or detoxification in FSTs, test whether expression can be induced by septic injury with bacteria and/or sublethal xenobiotic challenge with phenobarbital and to test if expression of immune response and/or xenobiotic metabolism genes is colony or caste dependent.

## Results

### 1. Constitutive and induced gene expression

**Figure 1:**
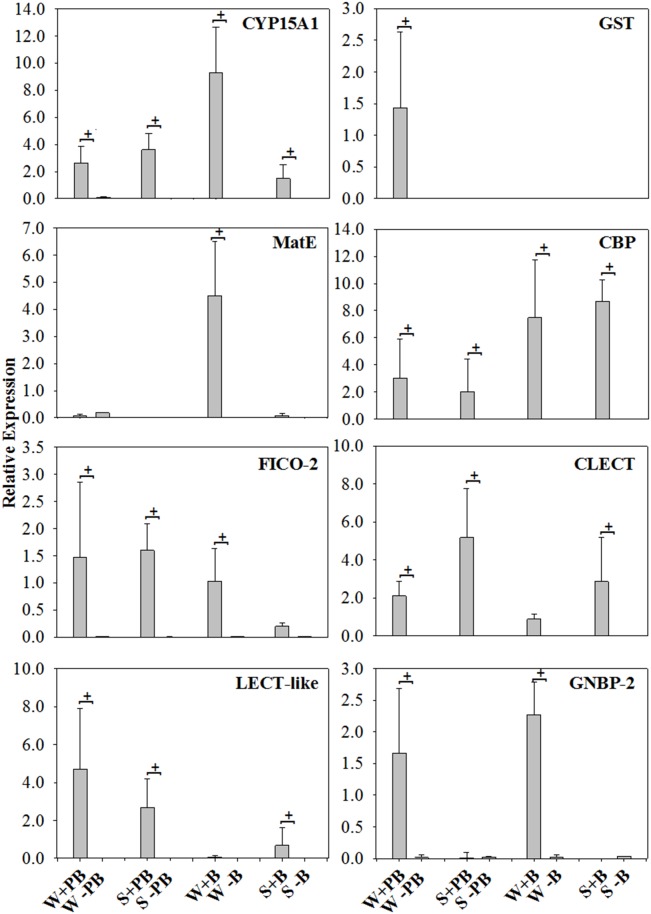
Constitutive and induced expression of target gene amplicons. The y-axes denote relative expression in R units averaged over three colonies (note that the ranges of y-values vary across graphs). The x-axes denote caste (W = worker, S = soldier) and treatment type (+PB = fed with 0.5% Phenobarbital, +B = septic injury with *E. coli/P. termitis* and -PB/-B = untreated control showing constitutive expression). ‘Missing’ columns have a relative expression of<0.01. + marks significant induction. There was no induced expression observed for either CYP450 or DSCAM (not shown).

Examined in this study were a total of 10 genes putatively involved in immunity, and/or detoxification in termites (Table 1). Constitutive (untreated controls) expression of target gene transcripts was rather low, overall (mean R = 0.014, SD = 0.163, Table S1). All but two target genes were inducible, either with a sublethal dose (0.5%) of the xenobiotic phenobarbital (PB) (mean R = 2.014, SD = 1.379) or septic injury with non-pathogenic bacteria (*Escherichia coli/Pilibacter termitis*) (mean R = 2.668, SD = 1.988) or both treatments (Table S1, Figure 1). Only one of the cytochrome P450 genes (CYP450) and the gene for Down syndrome cell adhesion molecule (Dscam) were not induced by either treatment. Most genes were inducible by both treatments. The genes inducible by xenobiotic and septic injury/immune challenge encoded for CYP15A1, Chitin-binding protein (CBP), two lectin-related proteins (LECT-like, CLECT), the Ficolin-2 precursor (FICO-2) and the gram-negative binding protein 2 (GNBP-2). The gene encoding for glutathione S-transferase (GST) was exclusively induced by 0.5% PB treatment, while the gene for the Multi antimicrobial extrusion protein (MatE) was induced predominantly with the *E. coli/P. termitis* treatment.

**Table 1:** Descriptions of target and reference genes.

Gene	Abbrev.	Speciesof Origin	Top Matches in GenBank,Species of Origin	PutativeFunction	PrimerSequence (5′-3′)
		(Accession No.)	(Accession No. for Nucleotideor Peptide Sequence)		
**Putative xenobiotic metabolism related genes**					
CytochromeP450 15A1	CYP15A1	*Coptotermes* *formosanus*	CYP151A, *Reticulitermes* *flavipes*(ACN93795)	Epoxidase,	ATTTGTCACTCGCCTTGGTC
		FK835449	CYP151A, *Diploptera punctata*(AAS13464)	juvenile hormonesynthesis	AAAGTTGCCATACGTGGAGG
CytochromeP450	CYP450	*Coptotermes* *formosanus*	CYP314a1, *Drosophila* *melanogaster* (Q9VUF8.3)	Ecdysone 20-monooxygenase	ACTGGTTTGTAGTCGGTGCC
		FK833823	CYP12A2, Musca domestica(O18635.1)	Monooxygenase,oxidoreductase	TTAGCACCGAGAGACGTTCA
Glutathion-S-Transferase	GST	*Hodotermopsis* *sjoestedti*	Glutathione S transferase,*Blattella germanica*	Metabolism oflipophilic toxins,	GTTTCAAGCCTCGCTTTTGAC
		DC239424	(FJ855500.1)	excretion ofxenobiotics	CGTCACTGTGAGACTGCCAT
Multi antimicrobial extrusion	MatE	*Coptotermes* *formosanus*	MatE, *Trichonomonas vaginalis*(XP_001314814.1)	Sodiumantiporter,cationic efflux	TTGACACAACACCTTTCGGA
protein		FK833694		transport pump,excretion oforganic cations	GCCGTGTTTGAGGATGAAAT
**Putative** **Immune** **related genes**					
Chitin bindingprotein	CBP	*Coptotermes* *formosanus*	chitin-binding protein,*Holotrichia oblita*(AFD28282.1)	Peritrophin A-typechitin-bindingprotein	CGGGAGGACTGCTAGAACTG
		FK832766			TGAGGGAACTCCCTGTTCAC
Downsyndrome	DSCAM	*Coptotermes* *formosanus*	Downsyndrome celladhesion molecule	immunglobulin-like fibronectin,	CCGTACCATTGACGGAGAGT
cell adhesionmolecule		FK835436	*Harpegnathos saltator*(EFN81146.1)	cell recognitionand adhesion,	CACTTGTGTTGCCCGTAATG
				axon guidancereceptor in insects	
Ficolin-2precursor	FICO-2	*Coptotermes* *formosanus*	Ficolin-2 precursor, *Pediculus* *humanus corporis*,(XP_002430827.1)	Antigenrecognition,carbohydrate binding,	CTCCGTCGGACTCTCATCTC
		FK836944	scabrous protein-like, *Tribolium* *castaneum*, (XP_972571.1)	Enhancesphagocytosis andopsonization	GCGCTATAACCTGGGTCTCA
Lectin-relatedhemolymph	LECT-like	*Coptotermes* *formosanus*	Hemolymph lipopolysaccharide-binding protein, *Periplaneta* *americana* (P26305.1)	*E. coli*Lipopolysaccharidebinding,	CTTCCCAGTGTCGAAAGCTC
Lipospolysaccharide bindingprotein		FK834461	Lectin-related regenectin,*P. americana* (BAA82265.1)	Tissueregeneration	TGGGTACCCCATAGGAGATG
C-type lectin	CLECT	*Coptotermes* *formosanus*	conserved hypothetical C-type lectin, *P. humanus corporis* (XP_002424769.1)	carbohydrate-binding, pathogenrecognition	AGGGTGAAAGAGGAGCAACA
		FK835521		innate immunity	TAAAAATCCCAACAACCCCA
Gram-negativebinding protein 2	GNBP-2	*Drepanotermes* *rubriceps*	gram negative bacteria binding protein 2 from different termite species	insect pathogenrecognition protein	CTTCCCAGTGTCGAAAGCTC
		DQ058934	(e.g., AAZ0893.1-AAZ08504.1, AEK64801.1, ADJ19004.1)	beta(1,3)-glucanase effector	TGGGTACCCCATAGGAGATG
**Reference** **genes**					
Cytoplasmicheat shockprotein 70	HSP	*Coptotermes* *formosanus*		ATP andnucleotide binding	AGCCTTGGCCACAACAGTGCAA
		FK835495			GGAGCAGGAGCAGGACCGACT
NADHDehydrogenasesubunit 4	NADH	*Coptotermes* *formosanus*		Mitochondrialelectron, sodiumion and	ACGAAGCAACCCATAACCACCAAGC
		FK833785		proton transport,ubiquinone activity	GGGCTCATGTTGAGGCTCCTGTT
Elongationfactor1-alpha	EFA	*Coptotermes* *formosanus*		GTP binding,	GGCGGTCACATTTCTCCTTA
		FK834645		regulation oftranslationalelongation	CTGAACCACCCTGGTCAGAT

### 2. Effects of colony origin

Tests for variance between colonies and for interactions between colony and treatment or between colony and caste showed no significant effect of colony origin at any of the 10 target genes. Probabilities of colony variability and interaction ranked from 0.127 to 0.783. Thus, regardless of treatment or caste, R values were not significantly different among the three termite colonies.

### 3. Effects of treatment

In contrast to colony origin, treatment type (0.5% PB vs. *E. coli/P. termitis)* had a significant effect on the expression of each of the eight inducible target genes (Figure 1, Table S1). CYP15A1 was induced in both types of treatment; however, relative expression was significantly higher (F = 88.03, P<0.0001: PROC GLM) for the *E. coli/P. termitis* treatment as compared to the 0.5% PB treatment due to a strong upregulation of gene expression in workers after bacteria treatment. Similarly, GNBP-2 showed induced expression in both treatment types but with significantly higher expression (F = 28.08, P<0.0001) for termite workers injected with *E. coli/P. termitis* than for termites fed with 0.5% PB. Also, CBP was significantly more induced (F = 31.19, P<0.0001) in *E. coli/P. termitis* injected termites than in 0.5% PB fed termites. MatE was only induced (F = 32.37, P<0.001) in the *E. coli/P. termitis* treatment group, while gene expression after PB treatment was not different from that of the controls.

In contrast, GST was exclusively induced after 0.5% PB treatment (F = 3.53, P = 0.0312), while gene expression did not differ from controls after *E. coli/P. termitis* treatment. Although induced by both treatments, relative expression of LECT-like, CLECT and FICO-2 was significantly higher (F = 33.03, P<0.001; F = 61.72, P<0.0001; and F = 23.78, P<0.001, respectively) for termites fed with 0.5% PB than for termites injected with *E. coli/P. termitis*.

### 4. Effects of caste

Tests for differential expression showed that caste (worker or soldier) had a significant (P<0.05) effect on levels of expression in five of the eight inducible target genes (CYP15A1, MatE, GST, GNBP, and CLEC, Figure 1, Table S1). The gene expressing CYP15A1 was induced in both workers and soldiers, with a significant interaction (P = 0.032) of treatment and caste, due to the significantly higher (F = 34.79, P<0.0001) expression in workers as compared to in soldiers. Constitutive expression of CYP15A1 was also significantly increased (F = 213.65, P<0.001, PROC GLM) for untreated workers as compared to untreated soldiers.

Constitutive expression of MatE was significantly higher (F = 40.16, P<0.0001) in untreated workers than in untreated soldiers as well. Furthermore, MatE was only induced in workers by septic injury. The Gram-negative bacterial binding protein (GNBP-2) was, similarly, almost exclusively induced in workers in both treatments (F = 121.23, P<0.001). GST was also exclusively induced in workers, but only after challenge with 0.5% PB. The C-type lectin gene (CLECT), was the only target gene which showed significantly higher (F = 47.53, P<0.001) expression in soldiers than in workers.

Caste had a marginal effect on the inducibility of Ficolin-2 (FICO-2) and chitin binding protein (CBP). These genes were induced both in workers and in soldiers, but FICO-2 was expressed marginally higher in the worker caste (F = 7.31, P = 0.058), while CBP’s expression was marginally biased towards soldiers (F = 8.10, P = 0.049). The second lectin related gene (LECT-like) also was induced in both castes, but showed no significant difference in expression levels as an effect of caste (F = 0.16, P = 0.692).

## Discussion

### 1. Effect of xenobiotic exposure and septic injury on expression of target genes

**Figure 2:**
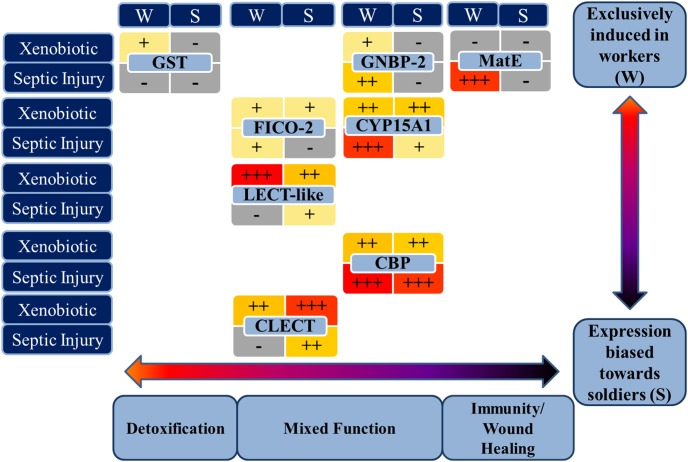
Graphic summary of caste- and treatment dependent inducibility and function of eight detoxification and immune-related genes of the Formosan subterranean termite. Treatment consisted of exposure to a xenobiotic substance (0.5% phenobarbital) or septic injury via bacteria injection. Relative levels of caste (workers vs. soldiers) dependent induced gene expression are indicated with +++ in red (>4 fold expression change compared to control), ++ in orange (2–4 fold expression change), + in yellow (<2 fold expression change) or – in grey (no significant induction). Genes are arranged from left to right according their putative function ranging from detoxification to immunity and from top to bottom according to their caste dependent expression.

Constitutive expression of all the investigated genes in FST workers and soldiers was low; however, eight out of the ten genes were inducible by one or both treatment types in one or both castes. The initial categorization based on the putative function of the ten target genes into genes associated with detoxification/xenobiotic metabolism and septic injury/immunity response was somewhat supported by the induction experiments using phenobarbital (PB) treatments and septic injury with bacteria, however, there were also some unexpected results (Figure 2).

The expression of the glutathione S-transferase (GST) and one of the cytochrome P450 genes (CYP15A1) as expected was upregulated in response to exposure with the xenobiotic PB. The barbiturate PB is used to treat epilepsy in humans and acts on GABA-gated chloride channels in the central nervous system to reduce excitatory synaptic response similar in activity to some termiticides, i.e., Fipronil [33].

Response elements and nuclear receptors responsible for induction of many cytochrome P450s and GSTs have been described in insects and shown to respond to several groups of xenobiotics, including PB [34]. In insects the GSTs are known to metabolize plant allelochemicals and insecticides, and high levels of GSTs are related to insecticide resistance. Exposure to PB has been previously shown to induce GST activity in cockroaches [35], *Drosophila* [36], [37], [38], *Aedes aegypti* [39], among others (reviewed in [40]). Upregulation of the expression of the GST gene in FST workers upon exposure to PB was therefore expected. The lack of upregulation in soldiers and the effect of caste on differential gene expression are discussed below.

Although, cytochrome P450 enzymes are largely ubiquitous and have common catalytic mechanisms, they are translated from a large family of genes with high sequence diversity, which explains their numerous functions. In insects, cytochrome P450s are known to play a role in detoxification of insecticides and xenobiotics (reviewed in [40]), but also in regulation of caste development via metabolism of hormones, like the juvenile hormone JH [41], [42], [43].

The closest matches for the gene product of one of the CYP450s in our study were a CYP15A1 of the cockroach *Diploptera punctata* (AAS13464.1, [41]) and a CYP15A1 pseudogene found in *R. flavipes* (ACN93795.1, [43]) with an identity of 51–54%, which lies within the range of cross-species alignments of translated CYP15s (41.5–72.5%, [43]). The product of CYP15A1 epoxidizes methyl farnesoate to juvenile hormone III in cockroaches [41], [44] and a similar function in JH biosynthesis was suggested in termites [43], [45]. Tarver et al. [43] showed that CYP15F1, which is closely related to the truncated CYP15A1 pseudogene, is involved in JH dependent caste differentiation. However, this study [43] did not investigate whether these two genes were functionally related.

Unlike other prominent cytochrome P450s, which metabolize a variety of pesticides due to their broad substrate and product specificity, CYP15A1 was shown to be highly specific in the silkworm [41]. The CYP15A1 gene in our study was the only target gene that was constitutively expressed and induced by both treatment types in workers and soldiers. The fundamental role in JH biosynthesis and caste regulation of similar genes in termites [43] may explain the level of constitutive expression observed in our study.

The inducibility by PB and septic injury, however, suggests that the CYP15A1 gene product has expanded functionality in terms of xenobiotic metabolism and immunity beyond its primary function as epoxidase catalyzing the last step in JH biosynthesis. While PB inducibility of CYP450s of the families 4, 6, 9, 12 and 28 in insects is well known, the only inducers for CYP15 family members so far reported were JH and terpene components from soldier head extracts suspected to act as caste regulatory primer pheromones [43]. Our study is the first to demonstrate inducibility of a member of the CYP15 family by a xenobiotic. However, the involvement of the CYP15 enzyme in actual xenobiotic metabolism and detoxification has to be confirmed in enzyme and bioassays. Similar to CYP15A1, another enzyme involved in JH metabolism (JH III epoxide hydrolase responsible for JH degradation) has been shown to be upregulated after PB exposure in several insect species ([46], and references therein).

A link between JH levels and innate immunity has been discovered in the silkworm *Bombus mori* [47], when JH treatment acted as an immune activator and increased the transcription products of six AMPs. We suggest that septic injury in our study upregulated CYP15A1 expression in termite workers and soldiers, which results in JH biosynthesis to provide increased JH levels as an immune activator. This route would constitute an immune pathway different but complementary to the Toll- and Imd pathways.

Overexpression of CYP450s and GSTs is often associated with insecticide resistance ([37], and references therein). However, the investigated CYP450s and GST genes were not constitutively expressed at elevated levels in Formosan termites and induction with PB led to a rather moderate spike in gene expression (less than 4 fold), which is in the range of susceptible insects [48]. Insecticide resistant strains usually show a much higher fold-change [49]. Although there is variability in tolerance to insecticides among termite colonies and species, to date, no resistance or treatment failure has been recorded.

The second cytochrome P450 gene in the study (CYP450) did not respond to treatment at all. CYP450 in our study was most closely related (48% identity) to *shade* (CYP314a1) from the Halloween gene family, an ecdysone 20-monooxygenase ecdysone hydroxylase involved in 20-hydroecdysone (molting hormone) synthesis in *D. melanogaster* [50]. CYP450 also showed considerable similarity to CYP12A2 from the house fly *Musca domestica*. A similar gene (CYP12A1) in the house fly was shown to be inducible by PB and to metabolize different insecticides [51]. Thus, we initially hypothesized that this gene would be most likely up-regulated in response to PB treatment and possibly involved in xenobiotic metabolism. However, this gene apparently lacks the response elements and/or transcription factors needed for PB inducibility [34]. Genome-wide studies in other insects showed that only a third of CYP genes are inducible by xenobiotics [52].

Similar to GST and CYP450 genes, we initially expected the gene for MatE to be inducible by the xenobiotic PB as MatE proteins have been described as multi drug resistance efflux proteins. They function as transmembrane pumps and are involved in the excretion of xenobiotics in vertebrates, detoxification of metals and secondary metabolites in plants [53] and mediating resistance to antibiotics in bacteria [54], [55]. It was somewhat unexpected that MatE expression was upregulated by septic injury and not by xenobiotic challenge in our study. The only evidence to date that MatE might be involved in antimicrobial defense is found in plant studies. Nawrath et al. [56] investigated a MatE gene homologue in *Arabidopsis* plants: expression was strongly induced by inoculation with an avirulent strain of *Pseudomonas syringae* and the authors concluded that this gene was likely involved in antimicrobial plant defense via the salicylic acid-dependent signaling pathway.

To our knowledge, MatE proteins and their function in insects have not yet been investigated. It is possible that the MatE gene in our study was directly involved in immune response in termites as indicated by the induced expression in FST workers injected with *E. coli* and *P. termitis*. However, it is more likely that MatE was upregulated as a consequence of the production of cytotoxic metabolites resulting from the activation of immune pathways by bacteria injection. For example, septic injury in insects activates the PPO pathway, which produces melanin, a phenolic biopolymer. Some melanin precursors are cytotoxic, which is advantageous for killing opportunistic invaders at the wound site [57]. However, metabolites might be inadvertently cytotoxic to the insect itself, especially since the PPO cascade is taking place in the open circulatory system and thus byproducts may accumulate in the hemolymph. MatE proteins play an important role in the removal of metabolic waste products and are believed to be a transport system for phenolic compounds [58]. Excessive melanin or its intermediaries from the PPO pathway might thus be extruded or sequestered by MatE in termites.

We initially assumed that the chitin binding protein (CBP) belongs into the category of immune related genes. The CBP in our study matched closest (31%) to a putative peritrophic membrane protein in a scarab beetle (*Holotrichia oblita*.), which contains carbohydrate and chitin binding domains. Invertebrates, such as shrimp, possess AMPs with chitin-binding capacity thought to be involved in bacteria phagocytosis, and perhaps antifungal activity and wound healing via chitin assembly [59]. Upregulation of CBP upon septic injury in workers and soldiers of Formosan termites suggests a similar involvement in immunity and/or wound healing via restoration of the cuticle.

However, PB treatment also induced CBP expression albeit at lower expression levels than bacteria challenge. A genome-wide analysis in the flour beetle, *Tribolium castaneum,* revealed that some proteins with peritrophin A-type chitin-binding domains were expressed in cuticle-forming tissues while others were associated with the peritrophic membrane and expressed in the midgut during feeding [60]. The peritrophic membrane is a barrier against pathogens and a sink for toxic substances [61], which would explain upregulation of certain peritrophins after ingestion of a xenobiotic like PB.

Similarly, the Ficolin 2 precursor (FICO-2) and the two lectins (CLECT, Lectin-like) in our study were assumed to be involved in immune defense enhancing phagocytosis and opsonization [62]. Ficolins are fibrinogen related proteins that act as PRRs by virtue of binding to carbohydrates on the surface of gram-negative and gram-positive bacteria [63]. This leads -at least in vertebrates- to the activation of the lectin complement pathway resulting in bacteria lysis. In insects, ficolins are not that well studied but the fact that their fibrinogen-like domain is conserved throughout the animal kingdom suggests that they are involved in ancestral forms of the innate immune system in invertebrates [64]–[66]. Genome-wide analyses and microarrays in *Drosophila* spp [15], [66] confirmed that fibrinogen-related domains are involved in immune response in insects. The Ficolin precursor in our study showed high similarity (78%) to a putative Ficolin-2 precursor of the human louse (*Pediculus humanus corporis*) and scabrous protein of *T. castaneum* (72%) and was upregulated by septic injury.

Both lectin related gene products in our study belong to the C-type lectin superfamily of calcium dependent carbohydrate-recognition proteins. C-type lectins play a variety of roles in adaptive and innate immunity as PRR, cell adhesion molecules and activators of the complement pathway [67]. Lectins in the hemolymph of cockroaches and lepidopterans participate in innate immune response via phagocytosis, encapsulation, nodulation and activation of the PPO pathway ([9], and references therein). Immunelectins vary in their specificity towards microbial epitopes as some show induction after Gram-negative bacteria injection only, while others bind to Gram-negative and Gram-positive bacteria as well as fungi [9].

The product of the LECT-like gene in our study showed 32% identity to both a hemolymph lipopolysaccharide (LPS)-binding protein [68] and regenectin [69] from the cockroach *Periplaneta americana*. The LPS binding protein had affinity for lipopolysaccharides on the cell membrane of some Gram-negative bacteria, but not others, suggesting high selectivity to certain oligosaccharide structures and thus specific immune recognition of selective pathogens [68]. Regenectin is involved in muscle formation and leg regeneration in cockroaches, but in contrast to LPS-binding protein, the transcription of its gene is not enhanced by bacterial infection [69]. Transcription of the LECT-like gene was upregulated in FST soldiers upon septic injury, which most likely reflects bacteria binding function and/or participation in tissue regeneration for wound healing.

The product of the second lectin related gene (CLECT) in our study was highly related to a conserved hypothetical protein in the human body louse (90% identity) and C-type lectin-like precursors from several insect species such as the flour beetle (89%), several mosquito species (≥86%), *Drosophila* spp. (≥86%), and social insects, i. e., ants, honey and bumble bees (≥88%). CLECT belongs thus to the immunelectins acting as PRR [9] that activate the PPO pathway. C-type lectins are also involved in sclerotization, i.e., exoskeleton hardening in arthropods [70]. Sclerotization is catalyzed by phenoloxidases and thus linked to activation of the PPO pathway. Furthermore, the calcium binding properties of C-type lectins are assumed to contribute to biomineralization of the cuticle by depositing calcium salts [70]. Thus, it was not surprising that CLECT was upregulated after septic injury similar to the LECT-like gene.

We were surprised, however, that the expression of the Ficolin precursor and both lectins was also induced by PB treatment. The majority of antiepileptic drugs such as PB show immunosuppressive effects in humans; however, under certain conditions, they can also stimulate the immune system [71]. Microarray analysis of gene expression revealed that *Drosophila* flies execute massive transcriptional response to PB treatment [72]. Not only were the detoxificication genes upregulated, but also genes involved in several metabolic and stress response pathways were affected. A nuclear receptor (DHR96) played a central role in regulating the response to PB. Middha and Wang [66] subsequently showed that some fibrinogen-related proteins are regulated by DHR96, thus linking expression of fibrinogen-like genes to PB exposure. This suggests a broader than originally assumed network of functions beyond innate immunity mediated by fibrinogen-like proteins in insects.

Gram-negative binding proteins are known in insects to sense pathogen invasion and trigger immune responses, i.e., the Toll and Imd pathways [73], and references therein) as well as the PPO cascade [74]. In termites, GNBP-2 has a double function as PRR that recognizes LPS (like lectins) and beta-1,3-glucans and as antifungal effector due to its beta-1,3-glucanase activity [21]. GNBP-2 is expressed in the salivary gland and by hemocytes and secreted via allogroming onto the cuticle and incorporated into nest and tunnel material presumably to suppress growth of fungal pathogens [21], [73].

Prior to our study, the role of GNBP in termites has been explored mostly in terms of antifungal but not antibacterial activity [73], [75], [76]. Our study showed involvement of GNBP-2 in defense against bacterial septic injury deduced from the fact that expression in workers doubled after bacteria injection. This result supports Bulmer et al.’s [21] *in vitro* binding assays that showed that GNBP-2 had affinity to Gram-negative bacteria.

While the upregulation of GNBP-2 upon immune challenge was expected, it was surprising that feeding on PB also induced GNBP-2 expression in workers. There is no straightforward explanation, but we may speculate that workers increase grooming of colony mates showing signs of PB exposure, which might indirectly lead to increased production of GNBP-2 in the salivary gland as protective secretion [21], [73]. Furthermore, PB acts as universal inducer for many genes in insects, not only CYP450s and GSTs but also for juvenile hormone epoxide hydrolases and for genes involved in carbohydrate metabolism, such as amylases [37]. GNBP-2 has a glucanase site that performs hydrolysis on glucosidic bonds and thus is a hydrolase involved in carbohydrate metabolisms. Like the increased transcription of other PRR immune genes upon xenobiotic exposure (see lectins and ficolin above) the regulatory and functional link between detoxification and immunity needs to be further investigated.

We initially hypothesized that the Down syndrome cell adhesion molecule (Dscam) was immune related. The gene in our study showed several putative conserved domains of the Immuneglobulin superfamily and fibronectin Type III similar to those in Hymenoptera and Diptera [77], which play a role in cell adhesion and wound healing in vertebrates. Insect studies showed that the Dscam receptor is involved in neural wiring and innate immunity [78]. Reduction of Dscam expression using mutants or RNA interference impaired phagocytosis of bacteria presumably due to reduced binding and opsonization of bacteria [79], [80].

Surprisingly, Dscam was not induced by septic injury (or by PB treatment) in our study. Dscam in the insect genome has many alternative exons and encodes tens of thousands of isoforms resulting from alternative splicing [77], [78]. Watson et al. [80] showed that some Dscam isoforms bound to *E. coli*, while others did not. There might be bacteria binding and non-binding isoforms in general, and/or binding is somewhat specific to epitopes of non-resident bacteria species, e. g., pathogens. The bacteria in our assay were native symbionts cultured from the termite gut, so the Dscam in our study might not be triggered.

### 2. Gene expression levels are mostly biased in favor of workers

Expression of five of the eight inducible target genes (CYP15A1, GST, MatE, GNBP-2, CBP and CLECT) was significantly and that of two additional genes (FICO-2, CBP) was marginally influenced by caste. Constitutive and/or induced gene expression was higher in workers in most of those differentially expressed genes except for CLECT, which was predominantly expressed in the soldier caste. In fact, three of the target genes (GST, MatE, and GNBP-2) were almost exclusively induced in workers.

In our study, higher expression of the “classic” detoxification genes such as members of the CYP450s (CYP15A1) and GST in the worker caste supports previous findings of higher enzyme levels in workers and higher tolerance against insecticides. Gatti and Henderson [6] and Gatti et al. [81] showed higher tolerance against organophosphates in workers attributed to a higher acetylcholinesterase activity in workers. Valles and Woodson [8] discuss their unpublished data which showed that soldiers of *C. formosanus* have only a fraction of detoxification enzyme activity compared to workers. Nevertheless, in a study of Osbrink et al. [7] soldiers of *C. formosanus* and *R. virginicus* in several colonies were more tolerant than workers to the majority of insecticides tested. This obvious variance among studies, species, enzymes and insecticides underlines the need for continued investigation of the relationship among gene expression, inducibility, metabolic enzyme production and activity, and insecticide susceptibility.

The major immune-related genes (GNBP-2, FICO-2) were induced and upregulated at higher levels after septic injury in workers than in soldiers. Soldiers might not rely primarily on the production of their own immune peptides for protection against pathogens. Soldiers have defense secretions, which in some species may have antifungal properties [23] and a higher degree of cuticle sclerotization than workers, which may aid as a mechanical barrier. Soldiers also may have antibacterial and/or antifungal activity via chitin assembly, wound healing and systemic protection related to increased CBP expression, which has been suggested for other highly sclerotized arthropods [59]. Sclerotization results from melanization and melanin plays an important role in innate immunity via wound healing and encapsulation of invaders. Melanin production during the PPO pathway also leads to toxic metabolites, such as quinones that could kill invaders [82]. Thus soldiers might have more non-protein related mechanical and biochemical defenses in form of sclerotization, melanin and its byproducts in addition to antimicrobial gland secretions. Soldiers could also receive passive immunization via secretions from workers. It has been shown that GNBP-2 is produced in the workers’ salivary gland and secreted to coat nest material and the cuticle [21] and that social transfer of immunity can occur [83]. Workers might overproduce GNBP-2 and other immune peptides and transfer them to soldiers via grooming.

CLECT was the only gene that was expressed at significantly higher levels in soldiers. C-type lectins are involved in mineralization and sclerotization of the cuticle [70]. Soldiers are more sclerotized than workers and thus upregulation of CLECT expression might be naturally facilitated and high levels of CLECT can be produced on demand. Although soldiers are likely to frequently get injured during colony defense, they did not upregulate the other immune proteins investigated in this study (GNBP-2 and Ficolin) to the same degree as workers. Consequently, the soldier caste might primarily rely on lectins, which they use naturally for exoskeleton hardening to support their innate immunity and complement passive transfer of immune proteins from workers secretions. Research into caste specific differences, concerning not only the level of immune defense but also the mechanisms and pathways used, might be of interest in future studies on social insects. The present study provides the foundation for this endeavor.

## Materials and Methods

### 1. Sample collection

Worker and soldier FSTs were collected in May 2009 from three field colonies in New Orleans, Louisiana, USA, an urban habitat in which this invasive species thrives: Colony 1 - City Park, adjacent to Tad Gromley Stadium at 1 Palm Drive; colony 2 - Louis Armstrong Park at 901 North Rampart Street and colony 3 - French Quarter at 300 Canal Street. No specific permissions were required for accessing these locations for sampling activities and no endangered or protected species were involved in the studies. Voucher specimens are deposited in the Louisiana State Arthropod Museum at the LSU Agricultural Center. Based on previous studies of FST colony size and flight distance [84]–[86], samples from these locations represented three independent colonies from the New Orleans population. Termites (∼500 workers and 500 soldiers) were collected from untreated inground monitoring station (Sentricon, Dow AgroSciences LLC: Indianapolis, IN) at each location and then transferred to plastic tubs (50×30×20 cm) lined with moist corrugated cardboard and maintained overnight in the laboratory at 25°C and 30% relative humidity (Intellus Environmental Controller, Percival: Perry, IA).

### 2. Determining sublethal treatment conditions for septic injury and phenobarbital challenge

Since bacteria invasion is generally associated with injury, and the responses to injury and infection are likely to be intricately connected, we decided to test the response to septic injury. To determine the appropriate sublethal treatment for inducing gene expression related to septic injury non-pathogenic bacteria, that were previously cultured from the guts of Formosan termite workers (gram-negative *Escherichia coli* [87] and gram-positive *Pilibacter termitis* [88] ATCC BAA-1030), were revived from glycerol stocks. *E. coli* was cultured under aerobic conditions in 1 mL of BHI broth for 1 day on a shaking incubator at 30°C, while *P. termitis* was grown anaerobically, using H_2_/CO_2_ BBL GasPaks (Becton Dickinson and Company: Franklin Lakes, NJ), in 1 mL of BHI broth at 30°C for 3 days. Sterile 28 gauge needles (Comfort Point: Nanaimo, British Columbia) were each soaked for approximately 60 seconds in a mixture of equal volumes of exponential growth phase *E. coli* and *P. termitis* (200 µL supernatant of each, placed in a 1.5 mL micro-centrifuge tube). Twenty workers and 20 soldiers from colony 1 were cold immobilized on a thermoelectric laboratory chill table (Bioquip: Gardenia, CA) and placed with their ventral surface exposed on a dissecting microscope stage (Leica MZ7.5 Stereomicroscope, Meyer Instruments: Houston, Texas). The abdomen was swabbed with 70% ethanol before being pierced through the intersegmental membrane of the 4^th^ and 5^th^ segments with the septic needle [18]. Following inoculation with bacteria, individual termites were isolated in 47 mm Petri dishes and kept at 25°C and 30% relative humidity (Intellus Environmental Controller, Percival: Perry, IA) for 48 hours. Twenty workers and 20 soldiers, which were not inoculated with bacteria, were held individually in separate dishes under the same conditions as controls. Mortality was determined as the inability for the termite to right itself after 30 seconds and was assessed for treated and untreated termites at 12, 24 and 48 hrs. Since no mortality was recorded at 24 hrs but 50% and 70% mortality in workers and soldiers, respectively, was recorded at 48 hrs, the 24 hr treatment was chosen as the sublethal treatment condition for target gene induction (Table 2). The 24 hr sublethal exposure time was consistent with rapid induction of innate immunity in insects (i.e., peptide synthesis in *Drosophila* [89] and in *P. spiniger* [18]).

**Table 2:** Mortality of FST workers and soldiers to determine sublethal treatment conditions.

Time (h)	Treatment	Mortality (%)	
		Workers	Soldiers
48	1% PB	100	100
24	1% PB	70	70
12	1% PB	50	70
48	0.5% PB	50	50
**24**	**0.5% PB**	**0**	**0**
12	0.5% PB	0	0
48	0.25% PB	0	0
24	0.25% PB	0	0
12	0.25% PB	0	0
48	0.125% PB	0	0
24	0.125% PB	0	0
12	0.125% PB	0	0
48	*E.coli/P. termitis*	50	70
**24**	***E.coli/P. termitis***	**0**	**0**
12	*E.coli/P. termitis*	0	0
48	Control	0	0
24	Control	0	0
12	Control	0	0

Termites were fed with specified concentrations of serially diluted phenobarbital (PB), injected with a 50∶50 mixture of *E. coli* and *P. termitis* or left untreated (Control). Sublethal treatment conditions (maximum dose and/or exposure time prior to observed mortality) for target gene induction are in bold print.

For the xenobiotic challenge we chose phenobarbital (PB, Sigma-Aldrich: St. Louis, MO), a universal inducer of a broad variety of detoxification genes in insects [36], [40]. Most studies test reactions to xenobiotics by topical application or ingestion. Since those would be the natural pathways of exposure in termites we used droplet feeding, which likely leads to ingestion and topical exposure via contact with the droplets and possibly transfer by grooming. To determine sublethal dose and time for PB treatments, 20 workers and 20 soldiers from colony 1 were each placed individually in a 47 mm Petri dish (Millipore: Billerica, MA). Five 2 µL droplets of 1% PB were pipetted directly onto the inside surface of each Petri dish. Droplets of 1% PB also were added to 20 Petri dishes which did not contain a termite as an evaporation control. All 60 dishes were sealed with parafilm and kept at 25°C and 30% relative humidity (Intellus Environmental Controller, Percival: Perry, IA) for 48 hrs. The PB-infused water droplets were monitored at 12 hr intervals. Level of evaporation was negligible over the 48 hr incubation period, and the PB droplets were consumed by workers and soldiers at approximately equal rate, confirming uptake.

We then used the droplet feeding assay to determine the maximum sublethal time and dose of PB treatment. Phenobarbital was serially diluted with distilled water (1%, 0.5%, 0.25% and 0.125%), and each concentration of PB was fed to 20 workers and 20 soldiers in 2 µl droplets as described above. Controls were fed distilled water only. Mortality was assessed as described above. Based on the results of this experiment (Table 2), the 0.5% PB treatment at 24 hrs was chosen as the sublethal treatment condition for target gene induction in both workers and in soldiers, which lies within the published time frame recommended for measuring PB- induced gene expression in insects ranging from 4 hrs in *Drosophila* [38] to 5 days in cockroaches [35].

### 3. Experimental design for septic injury and PB challenge

Following the established sublethal treatment conditions, 20 workers and 20 soldiers from each of the three FST colonies were fed 0.5% PB, 20 workers and 20 soldiers per colony were inoculated with the *E. coli/P. termitis* mixture and 40 workers as well as 40 soldiers per colony were left untreated (controls). Termites were kept in individual Petri dishes at conditions described above and flash-frozen at −80°C (Ultra low VIP, Sanyo Scientific: Wood Dale, IL) after 24 hrs. From each of the three colonies (biological replicates), five individuals of each caste (worker/soldier) and treatment (0.5% PB, septic injury with *E. coli/P. termitis* untreated control) were randomly chosen as technical replicates for quantitative real time PCR (qRT-PCR) reactions.

### 4. RNA isolation and cDNA synthesis

Total RNA was extracted from whole bodies of individual termites using the RNA Purification Microkit (QIAGEN, Valencia, CA). RNA concentrations were assessed at the 260/280 nm ratio of absorbance using a ND-1000 spectrophotometer (Nanodrop Technologies: Wilmington, DE). RNA samples were normalized to 10 ng per µl by adding RNAse free water and were then used as template for complimentary DNA (cDNA) synthesis via the iScript Kit (Biorad: Hercules, CA, USA) using a PTC-200 DNA Engine gradient thermocycler (MJ Research: San Francisco, California). The cDNA was normalized to 1,000 ng per µl and used as template for qRT-PCR.

### 5. Primer design for target and reference genes

We selected ten target genes putatively involved in immunity and xenobiotic metabolism from our Expressed Sequence Tag (EST) library of the FST [29], the literature and the NCBI database. Two ESTs matching to genes from Cytochrome P450 families [CYP15A1 (FK835449), CYP450 (FK833823)], and ESTs encoding for glutathione S-transferase [GST (DC239424)] and the multi antimicrobial extrusion protein [MatE (FK833694)] were assumed to be mainly involved in xenobiotic metabolism. Two ESTs encoding for lectin-related peptides [CLECT (FK835521), LECT-like (FK834461)], and those encoding for a Ficolin-2 precursor [FICO-2, (FK836944)], the Down syndrome cell adhesion molecule [Dscam (FK835436)], a chitin binding protein [CBP (FK832766)] and the gram-negative binding protein 2 [GNBP-2 (DQ058934)] are likely to play a role in pathogen recognition due to their putative cell adhesion function and were thus assumed to be immune related.

Primers for GST and GNBP-2 were already developed for a previous study that examined differential gene expression among FST queens and precopulatory females [29]. Three primer pairs were designed for each of the remaining seven target genes (PRIMER-BLAST software suite, NCBI) to have between 17 and 28 bases, with ∼50% to 60% GC content.

The EST library for FST [29] was first screened for reference genes used in previous studies (e.g., [90], [91]) for quantifying gene expression, i.e., Glyceraldehyde-3-phosphate dehydrogenase (FK834351), TATA-binding protein (FK835420), Elongation factor-1-alpha (FK834645) and Ubiquitin specific peptidase (FK831079). Next, the library was screened for genes most likely to be involved in cell maintenance and/or basal cell metabolism, i.e., “housekeeping” genes. Cytoplasmic heat shock protein (FK835495) and NADH dehydrogenase (FK833785) were selected, bringing the total number of putative reference genes to six. Table S2 lists the two to three primer pairs designed for each of the reference genes, along with their melting points, sequences and GC content.

Each primer pair designed for target genes was tested using qRT-PCR with cDNA from three untreated, randomly chosen workers from colony 1 as template. Primer pairs designed for reference genes were tested with cDNA template synthesized from three colony 1 workers and soldiers that were randomly chosen from each of the 0.5% PB treatment group, the *E. coli/P. termitis* treatment group and the untreated control group.

Amplifications of target and reference genes were performed using the IQ5 iCycler (Biorad: Hercules, CA, USA) in reactions each containing 5 µl cDNA, 25 µl SYBR-green Supermix (BioRad), 150 nm forward and reverse primer for each candidate or reference gene (see below), and 17 µl H_2_O for a total volume of 50 µl. The thermal protocol consisted of one cycle of 95°C for 3 min. followed by 40 cycles of 1) 95°C for 10 sec and 2) 58°C for 30 sec. Relative fluorescence units and Ct values (fractional cycle at which amplification reaches a detection threshold) were recorded at the end of each cycle by the iCycler software.

For each target gene, 1 of the 3 primer pairs was selected based on the following criteria [92]–[94]: 1) consistency across technical replicates (different individuals run on different plates), 2) target amplicon at 50 to 100 bp, 3) melting curve with a single peak and 4) reaction efficiency (E) ≥1.8. Primers for reference genes needed to pass all of the same criteria while also being consistent across treatment types (0.5% PB and *E. coli/P. termitis,* control) and among castes (workers and soldiers). Table S2 lists the ten target and three reference genes with their corresponding primer pairs which passed all of the above criteria.

### 6. Relative expression and tests for differential expression

LinRegPCR software [93] was used to calculate the individual efficiencies (E) for reactions, which were then averaged across the five technical replicates for each gene and treatment type. C_T_ values given by the iCycler qRT-PCR software were similarly averaged across technical replicates. For the three reference genes, geometric means (normalization factor: NF_n_, n = 3, [95]) of C_T_ and E values were calculated, and these geometric means were next used to calculate relative expression (R) of target genes. Relative gene expression levels (R) were calculated based on the methods described in [96], in which amplification efficiencies (E) for target and reference samples are not assumed to be equal [(R) = E*_target_* ^C^_T_^,*target (control-sample)*^ */*E*_ref_* ^C^_T_^,*ref (control-sample)*^] where *target* refers to the target gene under investigation and *ref* denotes the geometric mean of C_T_ and E values for the three reference genes. When calculating R values for constitutive expression, *control* refers to a non-template control, and *sample* refers to a biological control (i.e., untreated termites). However, when calculating R values for induced target gene expression, *control* refers to untreated controls, and *sample* refers to a treatment type (i.e., 0.5% PB or *E. coli/P. termitis*).

R values were compared between constitutively expressed and induced target amplicons within and among 1) three colonies, 2) three treatment groups (untreated control, 0.5% PB or *E. coli/P. termitis*), and 3) two castes (worker or soldier) via non-parametric, multivariate analysis of variance (PROC GLM, SAS/STAT Software 9.3, SAS Institute Inc., 2001). Furthermore, the GLIMMIX and RANK procedures were used to analyze non-normally distributed data points. The statistical model used was: gene = 1 to 10; expressed(gene) = treatment caste treatment*caste; random treatment*caste*colony, where “gene” refers to the 10 target genes, and “expressed(gene)” is the interaction of treatment, caste and colony. Differences in expression were considered significant when P<0.05.

## Supporting Information

Table S1 **Mean R values and standard deviations (SD) for target gene amplicons.**
(DOCX)

Table S2 **Primer pairs designed for six reference genes.**
(DOCX)
