# Supplementary material for: Effects of Caste on the Expression of Genes Associated with Septic Injury and Xenobiotic Exposure in the Formosan Subterranean Termite
Source: PLoS One. 2014 Aug 20;9(8):e105582. doi: 10.1371/journal.pone.0105582 (PMC4139394; doi:10.1371/journal.pone.0105582)
Supplement: Table S1 — Mean R values and standard deviations (SD) for target gene amplicons. (DOCX) [file pone.0105582.s001.docx]

**Table S1: Mean R values and standard deviations (SD) for target gene amplicons.**

Values were averaged across five technical replicates, for each gene and treatment type and for workers and soldiers from three Formosan subterranean termite colonies as biological replicates (W1, W2, W3 = workers and S1, S2, S3 = soldiers from colony 1, 2, 3, respectively). Induced target genes (expression significantly higher for treated termites than for untreated controls) are highlighted in bold numbers.
