# Supplementary material for: Effects of Caste on the Expression of Genes Associated with Septic Injury and Xenobiotic Exposure in the Formosan Subterranean Termite
Source: PLoS One. 2014 Aug 20;9(8):e105582. doi: 10.1371/journal.pone.0105582 (PMC4139394; doi:10.1371/journal.pone.0105582)
Supplement: Table S2 — Primer pairs designed for six reference genes. (DOCX) [file pone.0105582.s002.docx]

**Table S2: Primer pairs designed for six reference genes.**

Of the 15 tested primer pairs, only three pairs (bold) amplifying cytoplasmic heat shock protein 70, NADH dehydrogenase subunit 4 and elongation factor-1-alpha passed all of the selection criteria listed in Materials and Methods.
